# Combined 3D-QSAR and Docking Modelling Study on Indolocarbazole Series Compounds as Tie-2 Inhibitors

**DOI:** 10.3390/ijms12085080

**Published:** 2011-08-10

**Authors:** Yuanxin Tian, Jian Xu, Zhonghuang Li, Zhengguang Zhu, Jiajie Zhang, Shuguang Wu

**Affiliations:** School of Pharmaceutical Sciences, Southern Medical University, Guangzhou 510515, China; E-Mails: tyx523@163.com (Y.T.); woshixvjian@126.com (J.X.); lzhuang@fimmu.com (Z.L.); zzg@fimmu.com (Z.Z.); shuguang@fimmu.com (S.W.)

**Keywords:** Tie-2 kinase inhibitor, 3D-QSAR, CoMFA, CoMSIA, docking

## Abstract

Tie-2, a kind of endothelial cell tyrosine kinase receptor, is required for embryonic blood vessel development and tumor angiogenesis. Several compounds that showed potent activity toward this attractive anticancer drug target in the assay have been reported. In order to investigate the structure-activity correlation of indolocarbazole series compounds and modify them to improve their selectivity and activity, 3D-QSAR models were built using CoMFA and CoMSIA methods and molecular docking was used to check the results. Based on the common sketch align, two good QSAR models with high predictabilities (CoMFA model: *q*^2^ = 0.823, *r*^2^ = 0.979; CoMSIA model: *q*^2^ = 0.804, *r*^2^ = 0.967) were obtained and the contour maps obtained from both models were applied to identify the influence on the biological activity. Molecular docking was then used to confirm the results. Combined with the molecular docking results, the detail binding mode between the ligands and Tie-2 was elucidated, which enabled us to interpret the structure-activity relationship. These satisf actory results not only offered help to comprehend the action mechanism of indolocarbazole series compounds, but also provide new information for the design of new potent inhibitors.

## 1. Introduction

Tie-2, a kind of endothelial cell tyrosine kinase receptor, is expressed primarily by vascular endothelial cells and is required for embryonic blood vessel development and tumor angiogenesis [1]. Angiopoietin integrates with Tie-2 in the body to promote the Tie-2 receptor’s autophosphorylation, while the endothelial cells which express Tie-2 attract the cells around the vein to help the endothelial cells construct complete vessel walls, promote vascular remodeling and maturing, maintain the integrity of blood vessels and regulate their functions [2]. More and more data suggest that the inhibition of Tie-2 plays a vital part in curing cancer, and Tie-2 represents an important candidate for targeted therapy in cancer [3–5].

Recently, a novel series of indolocarbazole was reported as a kind of receptor tyrosine kinase inhibitor targeted to Tie-2 [6–9]. However, the QSAR focuses on indolocarbazole series compounds as Tie-2 inhibitors have not been reported. In our study, the 3D-QSAR models are constructed by CoMFA and CoMSIA methods on a training set of 80 indolocarbazole compounds as Tie-2 inhibitors. Comparative Molecular Field Analysis (CoMFA) and Comparative Molecular Similarity Indices Analysis (CoMSIA) are two commonly used three-dimensional quantitative structure–activity relationship (3D-QSAR) methods. It is a useful technique for understanding the pharmacological properties of studied compounds, because not only are the models visualized, but also the relationships between physical/chemical properties and pharmacological activity are represented in contour maps [10,11]. Combining with the docking study, the protein-ligand interactions were implicated. The models can help us to predict the biological activities of the series compounds with a change in the chemical substitutions and to provide some useful references for the design of new Tie-2 inhibitors. The theoretical results can offer some useful references for the design of new Tie-2 inhibitors as anticancer drugs.

## 2. Computational Methods

### 2.1. Data Set and Molecular Sketching

Except for some compounds with no activity or unclear activity, 80 indolocarbazole compounds from 4 references [6–9] are selected as the training set, among which 15 compounds are randomly chosen as the testing set (the testing set is marked by *). According to research practice, all original IC_50_ values (nmol/L) were converted to negative logarithm of IC_50_ (pIC_50_) and used as dependent variable in our 3D-QSAR study. The structure of these compounds and their activity values are listed in Table 1.

Among all the compounds in the data set, compound 27 was selected as template to construct other compounds because of its highest biological activity, and the computation is completed by SYBYL7.3 program package (*Tripos. Int.*) on a personal computer. Except for some special notes, default values are chosen to finish this work. The calculation can be defined as follows: after the construction of molecules, hydrogen and Gasteiger-Hückel charges were added to the compounds. Then their geometries are optimized by the conjugate gradient method in TRIPOS force field. The energy convergence criterion is 0.001 kcal/mol.

**Figure 1.**
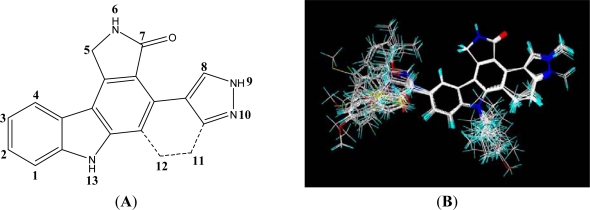
Superposition of compounds in the training and test sets using the common substructure-based alignment rules. (**A**) Common substructure-based alignment; (**B**) superposition of compounds in the training and test sets.

Structural alignment is considered as one of the most sensitive parameters in CoMFA and CoMSIA study. In our work, the most active compound 27, was used as a template for alignment, and the common fragment of 65 compounds is shown in Figure 1(A). The compound 27 was used as the template for alignment, and the rest of compounds were aligned on it [12]. This process was performed by Database Align of SYBYL 7.3. The aligned molecules were presented in Figure 1(B).

### 2.2. CoMFA and CoMSIA Study

In the CoMFA procedure, a 3D cubic lattice with a grid spacing of 2Ǻ was created automatically by the program to encompass all the aligned ligands. A default sp^3^-carbon probe atom with a van der Waals radius of 1.52 Ǻ and a charge of +1.0 was used to generate steric (Lennard-Jones 6–12 potential) field energies and electrostatic (Coulombic potential) fields with a distance-dependent dielectric at each lattice point. The computed field energy was truncated to 30 kcal/mol for both steric and electrostatic fields [12,13].

The CoMSIA procedure is similar to the CoMFA procedure. In this approach, five different similarity fields are calculated: steric, electrostatic, hydrophobic, hydrogen bond donor and hydrogen bond acceptor. The CoMSIA similarity index descriptors were derived using the same lattice box as that used in CoMFA calculations. These fields were selected to cover the major contributions to ligand binding. In CoMSIA fields, singularities were avoided at atomic positions because a Gaussian type distance dependence of each physicochemical property was adopted and thus no arbitrary cutoffs were necessary [14]. The attenuation factor was set to the default value of 0.3.

The Partial Least Squares (PLS) regression technique was used to construct a linear correlation between the 3D-field (independent variables) and the biological activity values (dependent variables). Cross-validations were performed by the leave-one-out (LOO) procedure to in which one compound was removed from the data set and its activity is predicted using a model built from the rest of data set [14,15]. It results in the cross-validation square correlation coefficient q^2^ and the optimum number of components (ONC). Then the optimum number of components was employed to construct 3D-QSAR models by non-cross-validations to obtain the conventional correlation coefficient *r*^2^, standard deviation SE and significant factor F. In order to speed up the analysis and reduce the noise, the column filter value of 2.0kcal/mol was set for non-cross-validations. The analysis procedure was performed by combing the bioactivity values (pIC_50_) and the corresponding field descriptor variation.

### 2.3. Predictive Correlation Co-efficient (*r*^2^_pred_)

In order to assess the predictive abilities of the CoMFA and CoMSIA models, the pIC_50_ values of an external test set composed of fifteen compounds were predicted. Furthermore, the predictive correlation coefficient *r*^2^_pred_ was calculated by the formula shown below.

$$r_{\text{pred}}^{2}=(\text{SD}-\text{PRESS})/\text{SD}$$

where SD is the sum of the squared deviations between the biological activities of the test set compounds and the mean activity of the training set compounds, and PRESS is the sum of squared deviations between experimental and predicted activity values of the test set compounds [16,17].

### 2.4. Molecular Docking

In order to determine the appropriate binding conformations of these compounds and check the main factors affecting the activity from the 3D-QSAR models, docking study for all compounds was performed with the Surflex-Dock program from Sybyl7.3. The Surflex-Dock uses an empirical scoring function and a patented search engine to dock ligands into a protein’s binding site [18]. The scoring function considers the four terms, including the hydrophobic complementarity, polar complementarity, entropic terms and solvation terms. Protomol, an idealized representation of a ligand that makes every potential interaction with the binding site, was used to guide molecular docking. In our study, the protomol was established guiding by the ligand in the protein. The X-ray crystal structure of Tie-2 was retrieved from the RCSB Protein Data Bank (PDB entry code: 3l8p). At the beginning of the docking, all the water and ligands were removed and the random hydrogen atoms were added. Then the receptor structure was minimized in 10,000 cycles with Powell method in sybyl7.3 [19]. The surface of protein was calculated with a MOLCAD program. Other parameters used in docking were default, except for those explained.

## 3. Results and Discussion

### 3.1. 3D-QSAR Models

Two QSAR models are established from CoMFA and CoMSIA analysis and the statistical parameters derived from the experiment are listed in Table 2. These parameters demonstrate that both QSAR models obtained are of high degree of confidence and strong predictive ability. The CoMFA model had a high cross-validated square correlation coefficient *q*^2^ (0.823) with an optimized component of 8, which suggests that the model is reliable and predictive, for *q*^2^ is greater than 0.5 indicating a model with good predictability [18–20]. The non-cross-validated square correlation coefficient *r*^2^ is 0.979(*r*^2^ is closer to 1 indicates better linear relationship) with a low standard error estimate (SEE) of 0.114, and F value of 319.429. Contributions of steric and electrostatic fields were 0.527 and 0.473, indicating that the steric interaction of the ligand with the receptor may be a more important influencing factor for the anticancer activity.

Compared with the CoMFA model, the CoMSIA model is a bit poor, but it is also a good model with high predictability (*q*^2^ value of 0.804 for eight components and a conventional *r*^2^ value of 0.967 with a SEE of 0.141, *F* value of 207.935, shown in Table 2). The *r*^2^_Pred_ value of 0.935 also shows that the predictive ability of the model are good, as does the CoMFA model with the *r*^2^_Pred_ value of 0.948. The contribution of five fields: steric, electrostatic, hydrophobic, hydrogen-bond donor and hydrogen-bond acceptor, were 0.121, 0.236, 0.160, 0.225, and 0.259, respectively.

**Figure 2.**
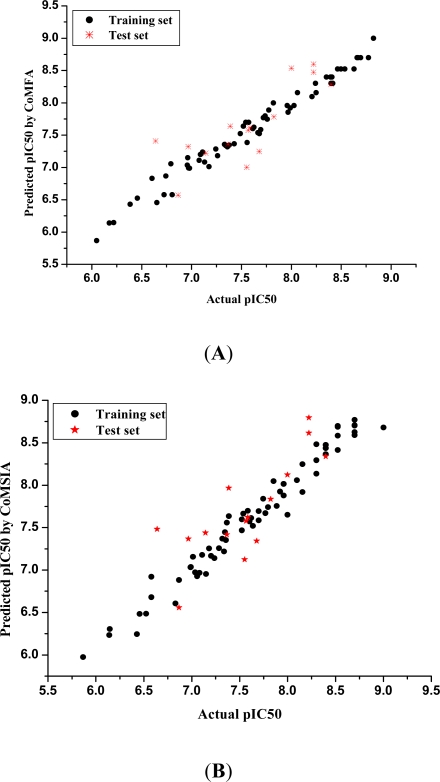
Graph of actual versus predicted pIC_50_ values of the training set and the test set molecular using the CoMFA model (**A**) and CoMSIA model (**B**).

The actual and predicted pIC_50_ values of the training set and the test set by two models are listed in Table 3, and the linear relationship for the CoMFA and CoMSIA analysis are shown in Figure 2 (a is the CoMFA model and b is the CoMSIA model), in which most points are evenly distributed along the line Y = X. It can clearly be seen that the predicted pIC_50_ values obtained from CoMFA and CoMSIA models are in good agreement with the experimental data.

### 3.2. Contour Analysis

The contour maps were used to display the fields around the molecules, and to rationalize where changes in each field probably affect the activity of the molecule. The models from CoMFA and CoMSIA were graphically interpreted through the stdev*coeff contour maps, which are plotted as the percentages of the contribution of CoMFA or CoMSIA equation. They show regions where variations of steric, electrostatic, hydrophilic, hydrogen-bond donor or acceptor nature in the structural features of the different molecules lead to an increase or decrease in the activity [20–22].

**Figure 3.**
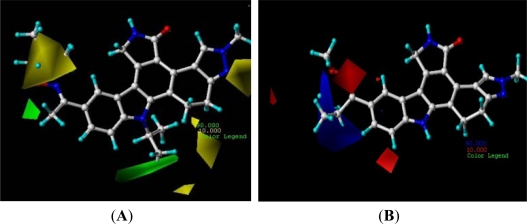
CoMFA Std*coeff contour maps illustrating steric, electrostatic field. Compound 16 was embedded in the map (**A**) while compound 5 was embedded in map (**B**). (**A**) Steric fields: green contours (90% contribution) indicate regions where bulky groups increase activity, while yellow contours (10% contribution) indicate regions where bulky groups decrease activity, and (**B**) Electrostatic fields: blue contours (90% contribution) indicate regions where electron-donating groups increase activity, while red contours (10% contribution) indicate regions where electron-withdrawing groups increase activity.

The contour maps of CoMFA are displayed in Figure 3. The steric field (A) is characterized by green and yellow contours, in which green indicates that increased steric is associated with enhanced activity and yellow indicated reduced activity. Compound 16 was selected as a reference molecule. There are green contours bellow the N-13 position, which suggested the suitable volume of alkyl at this position would increase the activity. The length of C3-C4 of N-alkyl substitution is probably suitable for improving the activity, shorter or longer lengths would decrease the activity. A bigger yellow contour beside the C-3 position and N-10 position shows that the more bulky substitutes in these areas will significantly decrease the biological activities. So, compared with the N-10 position alkynes substitutes (compound 77 and 78), the compounds with the methyl in the N-9 position (such as compounds 72 and 73) have bigger pIC_50_ values. Compound 16 has more potential than 15 because the *i*-tu is more bulky than *i*-Pr in the yellow area. This is satisfactory in accordance with the contour maps. The steric field (B) is characterized by blue and red contours, which indicates that the positive-charge groups and negative-charge groups would be favorable to the activity, respectively. As an electron-donating group, the isopropyl can decrease the positive-charge of the blue areas and decrease the activity, so compound 6 has the largest pIC_50_ value compared with compounds 1, 3 and 5. For another example, because the NHCO group is in the blue area, most of the compounds with phenyl urea have potential activity.

**Figure 4.**
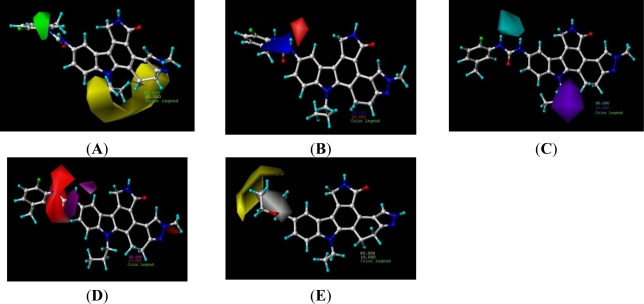
Std* coeff contour maps of CoMSIA illustrating steric, electrostatic, hydrophobic, hydrogen-bond donor and acceptor fields. Compound 16 was embedded in the map (**A**) while compound 4 was embedded in map (**B**). (**A**) Steric contour map: green contours refer to sterically favored regions while yellow contours refer to sterically disfavored regions. (**B**) Electrostatic contour map: blue contours refer to regions where electron-donating groups are favored while red contours indicate regions where electron-withdrawing groups are favored. (**C**) Hydrogen-bond donor contour map. The cyan (80% contribution) and the purple (20% contribution) contours indicate regions with favorable and unfavorable hydrogen-bond donor groups. (**D**) Hydrogen-bond acceptor contour map. The magenta contours (80% contribution) for hydrogen-bond acceptor groups increase activity; red contours (20% contribution) indicate the disfavored region. (**E**) Hydrophobic contour map. Yellow contours (85% contribution) indicate regions where hydrophobic substituents are favored, white contours (15% contribution) refer to regions where hydrophilic substituents are favored.

Compared with the CoMFA model, the CoMSIA model provides more information. The CoMSIA contour maps involve three parts: the electrostatic and steric field contours, the hydrophobic field contours, and the hydrogen-bond donor and hydrogen-bond acceptor field contours. The CoMSIA steric and electrostatic contour plots shown in Figure 4(A,B) are consistent to those of CoMFA. The big or small ramificate alkyl substituent of N-13 position would decrease the activity. The CoMSIA hydrophobic contour plot is shown in Figure 4E using compound 72. The yellow regions indicate hydrophobic substitutions will increase the activity of the compounds, while the white areas show that hydrophilic substitutions will increase activity. The compounds with NHCO groups at C-3 position have potential activity probably because the NHCO groups as a hydrophilic in the white area. If the NHCO group was replaced by C=O such as the compound-58 analogues, they also shows the activity. However if the NHCO group was replaced by C=N-O groups such as the compounds-1-4 analogues, the activities would be decreased because of the C=N in white area with less hydrophilic.

The hydrogen-bond donor and hydrogen-bond acceptor contour plots are shown in Figure 4 (C,D) using the compound 27. The blue regions highlight the hydrogen-bond donor’s contribution to the activity. The purple areas show the hydrogen-bond donor’s disadvantage in contributing to the activity. The hydrogen of the group NHCONH acted as hydrogen-bond donor would benefit the activity, however, if the hydrogen at the N-13 position is not replaced by alkyl, it would act as a hydrogen-bond donor in the purple areas, which can decrease the activity. This may explain why the compounds without N-alkylation at N-13 position have low pIC_50_ value. In the hydrogen-bond acceptor contour plot (Figure 4D), the red-purple and red regions represent those areas of favorable and unfavorable hydrogen-bond acceptor respectively. The C=O in the red regions shows it would decrease the activity. When the conformation was changed because of the substitution of phenyl (the C=O lie in the red-purple area), it could increase the activity, such as compounds 34 and 39.

### 3.3. Docking Analysis

In order to investigate the probable binding conformations between these indolocarbazole derivatives and the receptor, and to check the reliability of the established 3D-QSAR models, all studied inhibitors were docked into the active pocket of Tie-2. The crystal structure of Tie-2 was retrieved from the RCSB Protein Data Bank (PDB entry code: 3l8p). In order to validate the docking reliability, the ligand was removed from the active site and docked back into the binding pocket. The root mean square deviation (RMSD) between the predicted conformation and the actual conformation from the crystal structure of ligand was 0.609 Ǻ, which is smaller than the resolution of X-ray crystallography, 2.40 Ǻ. The results indicated that the parameter set for the Surflex-dock simulation was reasonable to reproduce the X-ray structure. Therefore the simulation method and the parameter set could be extended to study the binding conformations of the other inhibitors.

After all molecules were aligned according the method mentioned above, we found most of the molecules adopted a similar binding conformation to the potent inhibitor and had same orientation with each other. However, some inhibitor with low activity exhibited different poses and influenced the alignment based on docking, such as compound 19, 48, *etc*. The high quality 3D-QSAR models based on docking alignment could, unfortunately, not be constructed. A docking study showed that if the group of C-3 position is not enough to match to the sub-hydrophobic pocket composed of Asn887, Leu888, Leu876, Leu985, Asp982, Phe983, the molecule might be have much more orientation because of the large binding pocket of Tie-2. Most compounds had the same interaction with Tie-2, especially those with high activity, we still could acquire enough information to demonstrate our 3D-QSAR models. The following statements are the results of our docking studies, which are the complement of 3D-QSAR studies for drug design.

**Figure 5.**
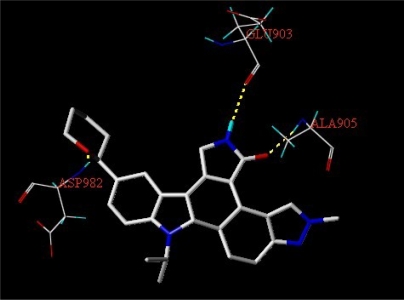
The binding mode between compound 73 and the Tie-2 acceptor (PDB: 3l8p). Key residues and hydrogen bonds are labeled.

The compounds 27 and 73 were selected for further detailed analysis. The best possible interacting model of compound 73 with Tie-2 and the main residues involved in the interaction were generally depicted in Figure 5. The O of C=O at C-7 position acted as a hydrogen-bond acceptor and formed a H-bond with the –NH of the Ala905 residue similarly to the hydrogen bond formed by O from epoxy cyclohexyl and the –NH of Asp982. The hydrogen of N-6 position served as the hydrogen donor by forming a H-bond with the C=O of the Glu903 residue. Docking study displayed almost compounds can form two hydrogen bonds: the O atom of C=O with H of –NH from Ala905 residue and the H of –NH with the O of C=O from Glu903. If the substitutes of C-3 position are not epoxy cyclohexane but urea dual or furan, the atom O generally acts as a hydrogen-bond acceptor and interacts with the amide acid of Tie-2.

**Figure 6.**
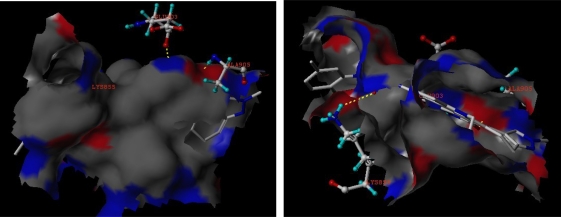
The MOLCAD hydrogen-bonding surface of ATP pocket of Tie-2 within the compound 27. There are only three colors: red (hydrogen donor, low electronegativity), gray (no hydrogen donor or acceptor) and blue (hydrogen acceptor, high electronegativity).

The knowledge of hydrogen-bonding sites on a molecular surface is a powerful tool for docking studies. Ligands can be docked to proteins by matching the patterns displayed on the surface. The surface is divided into three kinds of regions: hydrogen-bonding donating, hydrogen acceptor and the rest of the molecule. Figure 6 displayed the hydrogen bonding surface of active site of Tie-2 with in the compound 27. The observations obtained from Figure 6 are in agreement with that of CoMSIA hydrogen-bond donor and acceptor contour.

**Figure 7.**
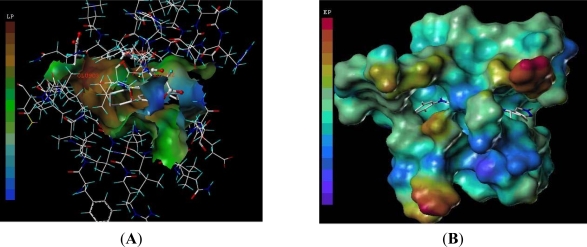
The MOLCAD lipophilic potential surface (**A**) and electrostatic potential surface (**B**) of ATP-binding site of Tie-2 within compound 27. The color ramp for LP ranges from brown (highest lipophilic area of the surface) to blue (highest hydrophilic area). The color ramp for EP ranges from red (most positive) to purple (most negative).

Figure 7 demonstrated the lipophilic potential surface (A) and electrostatic potential surface (B) of ATP-binding site of Tie-2 within compound 27. The methyl at N-9 position which is matched to the hydrophilic area is favorable to the activity. If the substitutes of 3-position is the flexible hydrophilic group which linked the hydrophobic phenyl substitutes, it will enhance the activity. The flexible bond may rotate to fit the hydrophobic pocket. Therefore the compounds-20-38 analogues have bigger pIC_50_ value. The conclusion from the lipophilic potential surface was satisfied according to the corresponding CoMSIA hydrophobic contour map. The compound 27 was docked into the ATP–binding site, the red color shows the electron-withdrawing zone and purple color shows electron-donating zone. In Figure 7(B), the R3 position was found in a yellow area.

### 3.4. Design for New Inhibitors

Based on the analysis of the structure-activity relationship and the docking studies, a series of novel compounds were designed as the active tie-2 inhibitor. These compounds were aligned to the database using compound 27 as a template and the theoretical pIC_50_ values were predicted by the CoMFA and CoMSIA models. They also were docked into the pocket of Tie-2. The chemical structure and the predicted pIC_50_ value so as to the dock score were shown in Table 4. The high predicted activity and the better dock score showed that they would be a potent inhibitor to Tie-2 in the future.

## 4. Conclusion

In this study, the 3D-QSAR study and molecular docking were carried out not only to construct highly accurate and predictive 3D-QSAR models, including the CoMFA (*q*^2^ = 0.823, *r*^2^ = 0.979, SEE = 0.114) and CoMSIA (*q*^2^ = 0.804, *r*^2^ = 0.967, SEE = 0.141), but also to explore the interaction mechanism between the indolocarbazole compounds and Tie-2. The results from the combined 3D-QSAR and docking study are: (1) The length of alkyl chain of N-13 position up to C3-C4 is favorable to the anticancer activity as larger or smaller alky cannot match to the pocket of active site of Tie-2; (2) The C-3 position suggested hydrophobic flexible group linked with the hydrogen-donor substituent are favored to the binding, which fits to the hydrophobic pocket of active site. Moreover, the hydrogen-donor group may form the hydrogen bond with the residues from Tie-2 through the rotation of flexible bonds. If the group is electron-negative at this position, the activity would be enhanced; (3) The methyl at N-9 position is more favorable than at N-10 position, because the bulky group in N-10 position may decrease the activity. The 6-position N-H and the 7-position C=O are requirements for the biological activity because of the hydrogen bonds and hydrophilic. The results from VEGFR2 did not conform to the regular, expected statistic for a kind of dual VEGFR2 and Tie 2 inhibitor and unfortunately the 3D-QSAR models could not be obtained. We can modify the indolocarbazole structures which were potent to VEGFR2 in order to improve the activity to Tie-2 based on the information from the QSAR models and docking and design new potent dual inhibitors to Tie-2 and VEGFR2.

## Figures and Tables

**Table 1.** Molecular structures of training and test set and their inhibitory activities (pIC_50_).

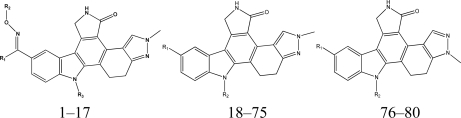				
**NO**	**R_1_**	**R_2_**	**R_3_**	**pIC_50_(nM)**
1	H	Me	H	6.83
2	H	Et	H	6.58
3	Me	Me	H	7.15
4	*i*-Pr	Me	H	6.58
5	*i*-Pr	Et	H	7.04
6	Me	*i*-Pr	H	7.55
7	Me	Me	Pr	7.52
8	Me	Me	*i*-Pr	7.01
9	Me	Me	*i*-Bu	7.52
10	Me	Me	*n*-Bu	6.97
11	Me	Et	*i*-Bu	7.60
12	Me	*i*-Pr	*i*-Bu	7.28
13	Me	*i*-Bu	*i*-Bu	7.64
14	Me	Et	*i*-Pr	7.58
15	Me	*i*-Pr	*i*-Pr	7.18
16	Me	*i*-Bu	*i*-Pr	7.34
17	Me	*i*-Bu	Et	7.35

**NO**	**R_1_**	**R_2_**	**pIC_50_(nM)**
18	NH_2_	CH_2_CH(CH_3_)_2_	7.32
19	NH_2_	CH_2_CH_2_CH_2_CH_3_	6.64
20	4-OMe–phenyl NHCONH	CH_2_CH_3_	8.40
21	4-OMe–phenyl NHCONH	CH_2_CH_2_CH_3_	8.70
22	4-OMe–phenyl NHCONH	CH_2_CH(CH_3_)_2_	8.52
23	4-OMe–phenylNHCONH	CH_2_CH_2_CH_2_CH_3_	8.15
24	4-SMe–phenylNHCONH	CH_2_CH_2_CH_3_	8.52
25	4-NMe2–phenylNHCONH	CH_2_CH_2_CH_3_	8.22
26	4-Me–phenylNHCONH	CH_2_CH_2_CH_3_	8.52
27	2-*F*-5-Me-phenylNHCONH	CH_2_CH_2_CH_3_	9.00
28	2-*F*-5-CF_3_-phenylNHCONH	CH_2_CH_2_CH_3_	8.40
29	PhenylNHCONH	CH_2_CH_2_CH_3_	8.30
30	Phenyl(Me)NCONH	CH_2_CH_2_CH_3_	8.70
31	3-OMe–phenylNHCONH	CH_2_CH(CH_3_)_2_	8.40
32	2-OMe–phenylNHCONH	CH_2_CH(CH_3_)_2_	8.10
33	4-*F*-phenylNHCONH	CH_2_CH_2_CH_3_	8.70
34	3-*F*-phenylNHCONH	CH_2_CH_2_CH_3_	8.70
35	2-*F*-phenylNHCONH	CH_2_CH_2_CH_3_	8.70
36	4-*Cl*-phenylNHCONH	CH_2_CH_2_CH_3_	8.22
37	2-*Cl*-phenylNHCONH	CH_2_CH_2_CH_3_	8.52
38	2-*Br*-phenylNHCONH	CH_2_CH_2_CH_3_	8.40
39	2-ThienylCONH	CH_3_	7.92
40	2-ThienylCONH	CH_2_CH(CH_3_)_2_	8.00
41	2-FuranylCONH	CH_2_CH_2_CH_3_	7.54
42	2-FuranylCONH	CH_2_CH(CH_3_)_2_	7.70
43	4-OMe–phenylOCONH	CH_2_CH_2_CH_3_	8.30
44	4-*F*-phenylOCONH	CH_2_CH_2_CH_3_	7.96
45	*i*-PrOCONH	CH_2_CH_2_CH_3_	7.96
46	EtOCONH	CH_2_CH_2_CH_3_	7.74
47	PrOCONH	CH_2_CH_2_CH_3_	8.15
48	H	H	5.87
49	H	*n*Pr	6.87
50	H	*i*-Bu	6.43
51	Ac	*i*-Pr	6.14
52	Ac	*i*-Bu	7.08
53	2-Thiophene-CO	H	6.87
54	2-Thiophene-CO	Et	7.37
55	2-Thiophene-CO	Pr	7.59
56	2-Thiophene-CO	*i*-Pr	7.62
57	2-Thiophene-CO	*i*-Bu	7.70
58	3-Thiophene-CO	*i*-Bu	7.20
59	2-Furan-CO	*i*-Bu	7.14
60	3-Cl-Thiophene-2-CO	CH_2_CH(CH_3_)_2_	7.89
61	3-Br-Thiophene-2-CO	CH_2_CH(CH_3_)_2_	7.82
62	3-Me-Thiophene-2-CO	CH_2_CH(CH_3_)_2_	8.30
63	4-Me-Thiophene-2-CO	CH_2_CH(CH_3_)_2_	7.37
64	3-Furan-CO	*i*-Bu	7.24
65		H	7.36
66		*c*-Pentyl	7.39
67		*c*-Hexyl	6.99
68	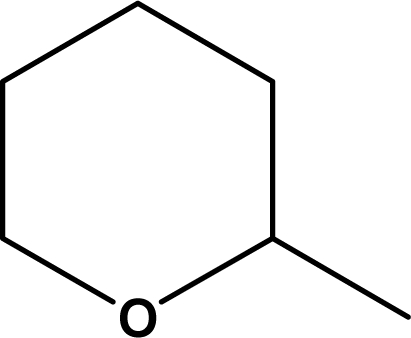	CH_2_CH_2_OEt	7.77
69		(CH_2_)_2_OH	7.57
70		(CH_2_)_3_OH	7.80
71		Me	7.39
72		Et	7.85
73		*i*-Pr	8.00
74		*i*-Pent	7.68
75		*n*-Pent	7.11
76		H	6.15
77		Et	7.06
78		*i*-Pr	6.99
79		*c*-Pentyl	6.52
80		(CH_2_)_2_OH	6.46

**Table 2.** Results of CoMFA and CoMSIA Models.

**PLS Statistics**	**CoMFA**	**CoMSIA**
*q*^2^^a^	0.823	0.804
*r*^2^^b^	0.979	0.967
ONC ^c^	8	8
SEE ^d^	0.114	0.141
*F* value^e^	319.429	207.935
*r*^2^_pred_^f^	0.948	0.935
Field Contribution(%)		
Steric	0.527	0.121
Electrostatic	0.473	0.236
Hydrophobic	–	0.160
H-bond Donor	–	0.225
H-bond Acceptor	–	0.259

a cross-validated square correlation coefficient;

b non-cross-validated square correlation coefficient;

c optimal number of components;

d standard error of estimate;

e value *F*-test;

f predictive correlation coefficient.

**Table 3.** The experimental pIC_50_ values(nM), predicted pIC_50_ value (Pred.) and their residuals (Res.) of the indolocarbazole derivatives training and the test set molecules (labeled by *).

**Compd. No.**	**Experimental**	**CoMFA**		**CoMSIA**	

		**Pred.**	**Res.**	**Pred.**	**Res.**

1	6.830	6.604	0.226	6.606	0.224
2	6.578	6.726	−0.148	6.679	−0.101
3	7.149	6.961	0.188	6.953	0.196
4	6.578	6.807	−0.229	6.921	−0.343
5	7.036	6.958	0.078	6.973	0.063
6 *	7.553	7.001	0.552	7.122	0.431
7	7.523	7.490	0.033	7.467	0.056
8	7.013	7.175	−0.162	7.157	−0.144
9	7.523	7.679	−0.156	7.597	−0.074
10 *	6.967	7.321	−0.354	7.366	−0.399
11	7.602	7.615	−0.013	7.571	0.031
12	7.284	7.243	0.041	7.258	0.026
13	7.638	7.520	0.118	7.519	0.109
14 *	7.585	7.615	−0.030	7.620	−0.035
15	7.180	7.261	−0.081	7.254	−0.074
16	7.337	7.345	−0.008	7.220	0.117
17	7.347	7.376	−0.029	7.447	−0.100
18	7.319	7.359	−0.040	7.370	−0.051
19 *	6.638	7.410	−0.771	7.481	−0.842
20	8.398	8.394	0.004	8.366	0.032
21	8.699	8.774	−0.075	8.589	0.110
22	8.523	8.496	0.027	8.415	0.108
23	8.155	8.249	−0.094	8.249	−0.094
24	8.523	8.537	−0.014	8.583	−0.060
25 *	8.223	8.472	−0.251	8.613	−0.390
26	8.523	8.628	−0.105	8.698	−0.175
27	9.000	8.823	0.177	8.680	0.320
28	8.398	8.351	0.047	8.475	−0.077
29	8.301	8.416	−0.115	8.484	−0.183
30	8.699	8.662	0.037	8.626	0.073
31 *	8.398	8.286	0.112	8.338	0.060
32	8.097	8.208	−0.111	8.059	−0.038
33	8.699	8.659	0.040	8.701	−0.002
34	8.699	8.694	0.005	8.707	−0.008
35	8.699	8.668	0.031	8.771	−0.072
36 *	8.223	8.596	−0.374	8.794	−0.572
37	8.523	8.465	0.058	8.689	−0.166

**Compd. No.**	**Experimental**	**CoMFA**	**CoMSIA**	**Compd. No.**	**Experimental**

		**Pred.**	**Res.**		

38	8.398	8.406	−0.008	8.436	−0.038
39	7.921	7.989	−0.068	7.925	−0.004
40 *	8.000	8.537	−0.537	8.121	−0.121
41	7.538	7.665	−0.127	7.664	−0.126
42	7.699	7.545	0.154	7.587	0.112
43	8.301	8.243	0.058	8.136	0.165
44	7.959	8.027	−0.068	8.014	−0.005
45	7.959	7.960	−0.001	7.878	0.081
46	7.745	7.758	−0.014	7.839	−0.094
47	8.155	8.063	0.092	7.919	0.236
48	5.867	6.049	−0.182	5.975	−0.108
49 *	6.867	6.571	0.296	6.557	0.310
50	6.429	6.384	0.045	6.244	0.185
51	6.139	6.177	−0.038	6.233	–0.094
52	7.081	7.132	−0.051	6.968	0.113
53	6.866	6.742	0.124	6.882	−0.016
54 *	7.366	7.360	0.006	7.416	−0.050
55	7.585	7.694	−0.109	7.669	−0.114
56	7.620	7.625	−0.005	7.614	0.006
57	7.699	7.572	0.127	7.694	0.005
58	7.201	7.089	0.112	7.168	0.032
59 *	7.143	7.219	−0.076	7.437	−0.294
60	7.886	7.775	0.111	7.755	0.131
61 *	7.824	7.782	0.042	7.834	−0.010
62	8.301	8.397	−0.096	8.293	0.008
63	7.366	7.428	−0.062	7.559	−0.193
64	7.237	7.111	0.126	7.139	0.098
65	7.356	7.330	0.026	7.354	0.002
66	7.387	7.557	−0.170	7.634	−0.267
67	6.991	6.971	0.002	7.037	−0.044
68	7.770	7.719	0.051	7.671	0.099
69 *	7.569	7.574	−0.005	7.577	−0.009
70	7.796	7.738	0.058	7.741	0.055
71 *	7.387	7.639	−0.252	7.966	−0.579
72	7.854	7.968	−0.114	8.049	−0.195
73	8.000	7.821	0.179	7.650	0.350
74 *	7.678	7.247	0.431	7.343	0.335
75	7.108	7.077	0.031	7.179	−0.071
76	6.146	6.219	−0.073	6.305	−0.159
77	7.056	6.794	0.262	6.926	0.130
78	6.987	6.979	0.008	7.032	−0.045
79	6.522	6.456	0.066	6.485	0.037
80	6.456	6.652	−0.196	6.484	0.028

**Table 4.** Chemical structure of newly designed compounds and their predicted pIC_50_ and the Surflex-dock total-scores.

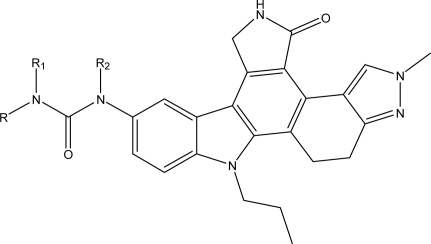						
**No.**	**Substituent**			**Predicted pIC _50(nM)**		**Total-Score**

	**R**	**R_1_**	**R_2_**	**CoMFA**	**CoMSIA**	
D1	2-F-5-Me-phenyl	CH_3_	H	9.024	8.380	13.35
D2	2-F-5-Me-phenyl	CH_2_CH_3_	H	8.966	8.508	14.02
D3	5-Me-phenyl	CH(CH_3_)_2_	H	8.906	8.519	11.88
D4	2-F-5-Me-phenyl	CH(CH_3_)_2_	H	8.951	8.515	11.18
D5	2-F-5-Me-phenyl	H	OH	8.953	8.896	11.54
D6	2-F-5-Me-phenyl	OH	OH	8.971	8.588	11.43
D7	2-F-5-Me-phenyl	OCH_3_	OH	8.947	8.572	11.31
D8	2-F-5-Me-phenyl	OH	CH_3_	8.786	8.250	12.36
D9	3-F-6-Me-2-pyridyl	H	H	8.769	8.655	9.69
